# Increased Mannosylation of Extracellular Vesicles in Long COVID Plasma as a Binding Target for *Galanthus nivalis* Agglutinin (GNA) Affinity Resin

**DOI:** 10.3390/ijms27135723

**Published:** 2026-06-25

**Authors:** Miguel A. Pesqueira Sanchez, Rosalia de Necochea Campion, Thomas Dalhuisen, Emily A. Fehrman, Pahul S. Chhabra, J. Daniel Kelly, Jeffrey N. Martin, Steven G. Deeks, Timothy J. Henrich, Michael J. Peluso, Steven P. LaRosa

**Affiliations:** 1 Aethlon Medical, Inc., San Diego, CA 92121, USA; slarosamd@aethlonmedical.com; 2 Department of Medicine, University of California, San Francisco, San Francisco, CA 94110, USA

**Keywords:** Long COVID, extracellular vesicles, glycosylation, microRNA, immunomodulation

## Abstract

There is no proven therapy for Long COVID, a post-acute condition characterized by persistent symptoms following SARS-CoV-2 infection. Extracellular vesicles (EVs) are emerging as mediators of disease pathogenesis through their molecular cargo. We investigated whether EV glycosylation is altered in Long COVID plasma and whether these vesicles can be selectively targeted using a glycan-binding affinity resin. Large (100–500 nm) and small (40–200 nm) EVs were isolated from post-acute COVID-19 plasma and analyzed by nanoparticle flow cytometry to assess surface glycosylation. Small EV capture assays were performed using *Galanthus nivalis* agglutinin (GNA) affinity resin. Plasma miRNA profiles before and after GNA treatment were evaluated using NanoString nCounter analysis, and potential downstream pathway effects were computationally inferred using validated miRNA–mRNA interactions and PROGENy. Mannose-positive large EVs were significantly increased in Long COVID compared to recovered controls (*p* < 0.05). GNA-mediated small EV capture correlated with mannose-positive EV abundance (r = 0.341, *p* < 0.05), and seven miRNAs were significantly reduced following treatment. Computational pathway analysis suggested modulation of key signaling pathways, including JAK-STAT, Estrogen, VEGF, and PI3K. These findings suggest a glycan-associated EV signature in Long COVID and support further investigation of lectin-based capture as a potential strategy to target vesicle-associated molecular cargo.

## 1. Introduction

Following the acute phase of SARS-CoV-2 infection, a subset of people experience Long COVID [1], a post-acute condition characterized by ongoing symptoms that may include fatigue, post-exertional malaise, dyspnea, chest pain, and troubling neuro-psychiatric problems including “brain fog” [2,3]. Despite its estimated prevalence—6–7% of the U.S. population [4], potentially hundreds of millions of people globally and its large burden (~1 trillion dollars per year [5])—management remains focused on symptom relief and no curative treatments are available.

Multiple pathophysiologic mechanisms have been proposed to contribute to the development of Long COVID. These include persistence of SARS-CoV-2 RNA and protein, reactivation of latent herpesviruses (e.g., Epstein–Barr Virus), immune dysregulation and autoimmunity, thrombosis, dysbiosis, and mitochondrial and tissue dysfunction [6,7]. Extracellular vesicles (EVs), small nanoparticles with a lipid bilayer approximately 30−1000 nm in diameter, released from all cell types and involved in cell-to-cell communication, have been implicated in the pathogenesis of Long COVID [8]. EVs have been found to contain viral components including the SARS-CoV-2 spike protein [9,10].

EV surface glycans, including high-mannose N-glycans, regulate vesicle biogenesis, biodistribution, cellular uptake, and immune interactions, making EV glycosylation a central functional feature and potential target for capture or diagnostics [11]. In acute COVID-19, there is evidence supporting a functional glycome mechanism in disease pathology, with glycan changes observed in immune effector proteins in both plasma and lung tissues [12]. Disease-associated alterations in EV glycosylation have also been described in other settings. Cancer-derived EVs can display distinct glycan profiles, including enrichment of high-mannose-type glycans that permit lectin-based EV recognition and capture [13]. In an infectious-disease model, EVs released from Plasmodium falciparum-infected human erythrocytes exhibited increased sialylated complex N-glycans compared with EVs from uninfected erythrocytes, and these glycans contributed to EV uptake by human monocytes [14]. These findings support the broader concept that disease-associated EV glycosylation can influence vesicle recognition, capture, and interactions with recipient immune cells. Notably, the Aethlon Hemopurifier^®^, which targets specific glycan structures, was shown to remove circulating SARS-CoV-2 virus, extracellular vesicles, and pathogenic miRNA cargo from critically ill COVID-19 patients [15]. The Hemopurifier^®^ is a plasmapheresis cartridge filled with *Galanthus nivalis* agglutinin (GNA) affinity resin, which binds mannosylated glycoproteins found on the surface of enveloped viruses and some EVs [16]. Persistent glycome remodeling of serum proteins in acute COVID-19 has been associated with disease severity and duration in multiple independent cohorts [17], raising questions about whether similar alterations persist in Long COVID plasma-derived EVs.

In this study, we measured the abundance and mannosylation characteristics of plasma-derived EVs from participants with Long COVID and compared them to EVs from individuals who had fully recovered after an episode of COVID-19. Our objective was to determine whether altered EV glycosylation composition is a feature of this illness. We describe the association between Long COVID EV mannosylation patterns and capture dynamics on the GNA affinity resin. Furthermore, we evaluated circulating plasma miRNA changes following GNA affinity resin treatment to identify miRNAs reduced in association with resin-mediated capture. Pathway inference analysis was then used to generate hypotheses regarding signaling networks potentially linked to these miRNA changes, including pathways involved in immune regulation and Long COVID-related biology. Together, these findings provide a framework for further evaluating mannose-enriched EV populations and GNA-based capture of glycosylated EV-associated plasma components in Long COVID. Since GNA affinity resin is incorporated into the Aethlon Hemopurifier^®^, this novel research provides a translational basis for future studies evaluating whether EV targeted extracorporeal capture can remove plasma components relevant to Long COVID pathology.

## 2. Results

### 2.1. Cohort Description

Clinical characteristics were similar across the 3 groups apart from higher BMI and greater number of symptoms in the LC groups (Table 1).

### 2.2. Plasma EV Characterization

**Figure 1:**
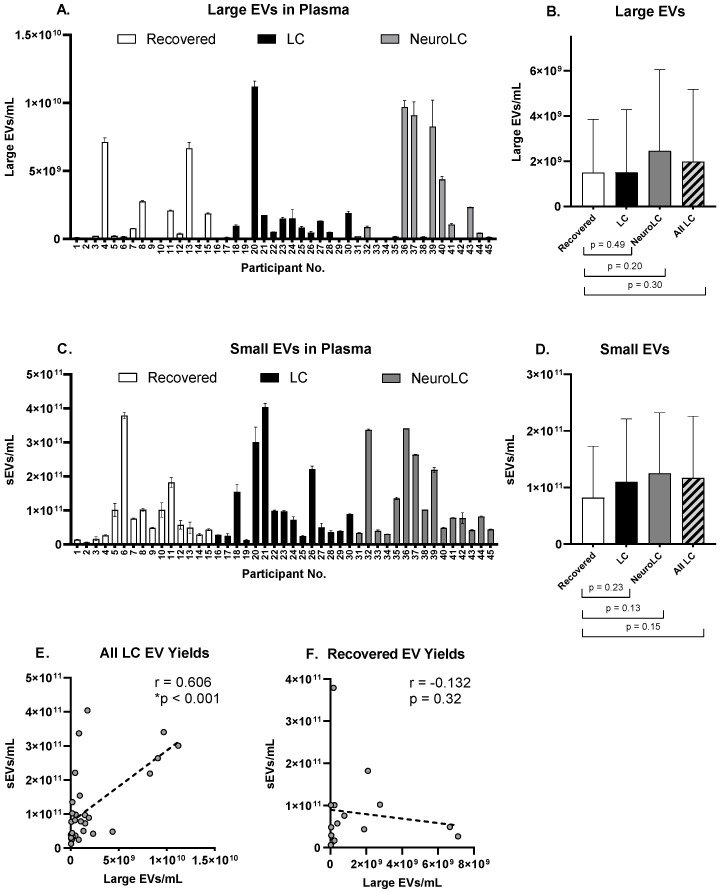
Plasma extracellular vesicle yields in the post-acute phase of COVID-19. (**A**) Large EV yields (100–500 nm). Data shown represents the average and SD of two technical replicates; (**B**) Average ± SD quantities of large EVs per symptom group (*n* = 15); (**C**) small EV yields (40–200 nm). Data shows the average and SD of two technical replicates; (**D**) Average ± SD quantities of sEVs measured in each symptom group (*n* = 15); (**E**) Correlation of large and small EV quantities isolated from All LC (r = 0.606, * *p* < 0.001, *n* = 30); (**F**) and from Recovered plasmas (r = −0.132, *p* = 0.32, *n* = 15).

Plasma large EVs (100–500 nm) displayed substantial variability in individual sample concentration (Figure 1A). On average, the NeuroLC plasma had 64% higher concentrations of these vesicles than LC and Recovered plasma; however, this difference was not statistically significant (Figure 1B). Small EVs (40–200 nm) isolated from plasma also displayed considerable individual sample variability (Figure 1C), with average quantities highest in the NeuroLC group, although this increase was not statistically significant either (Figure 1D).

When we examined the association between quantities of large and small EVs in All LC we detected a significant positive correlation between concentrations of these two EV subpopulations (Figure 1E). In contrast, no such association was observed among EVs from fully recovered participants as no relationship was detected among concentrations of large and small EVs isolated from Recovered plasma (Figure 1F). These findings suggest that relationships among EV subpopulations may differ in Long COVID plasma.

**Figure 2:**
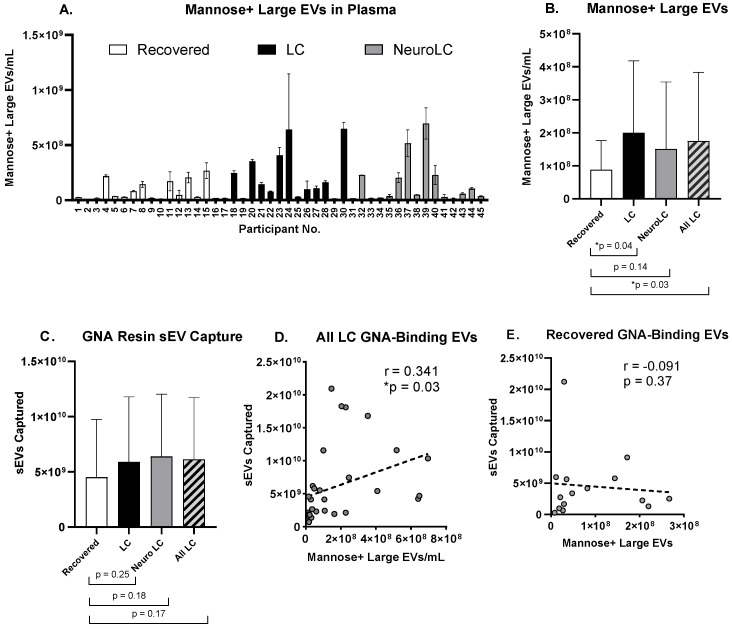
Increased mannose glycosylation in symptomatic plasma EVs improves GNA-based capture. (**A**) Abundance of mannose-positive large EVs (100–500 nm). Data shown represents the average and SD of two technical replicates; (**B**) Average quantities of mannose-positive large EVs measured in each symptom group (*n* = 15, except for All LC where *n* = 30). Comparisons to control (Recovered) group show statistical differences (* *p* < 0.05). (**C**) Average quantities of sEVs (40–200 nm) removed by GNA affinity resin treatment by symptom group (*n* = 15, except for All LC where *n* = 30) (**D**) Correlation between sEV capture on GNA affinity resin and mannose-positive EV abundance in All LC (r = 0.341, * *p* < 0.05, *n* = 30); (**E**) and in Recovered samples (r = −0.091, *p* = 0.37, *n* = 15).

In order to determine if there were differences in the glycosylation patterns of EVs in the distinct Long COVID symptom groups, we evaluated concentrations of mannose-positive large EVs (100–500 nm) in these plasma samples. We observed a large range of individual variability in the quantities of mannose-positive large EVs (Figure 2A), with average quantities two-fold significantly higher in All LC groups (Figure 2B). Although binding of fluorescently labeled GNA to mannose glycans on the surface of smaller EVs (<100 nm) did not produce a signal strong enough to quantify, capture of sEVs to the GNA affinity resin could be tracked. On average, we observed 31–42% increased sEV capture by GNA affinity resin treatment of All LC groups compared with the control (Recovered), although these differences were not significant (Figure 2C). A weak but significant positive correlation was observed between mannose-positive large EV quantities and sEV capture by GNA resin treatment in All LC samples (Figure 2D). In contrast, no association was found between mannose-positive large EV abundance and GNA resin capture of sEVs isolated from plasma of Recovered controls (Figure 2E).

### 2.3. Plasma miRNA Analysis

To characterize miRNA removed by GNA affinity resin treatment, we analyzed changes to miRNA content of All LC plasma samples (*n* = 20) after incubation with GNA affinity resin using NanoString nCounter technology. Of the 827 human miRNAs of biological relevance that can be evaluated using the off-the-shelf NanoString miRNA panel, 358 were detected in quantities above the background threshold. Approximately 36% of these miRNAs (128/358) were found to be significantly removed by GNA affinity resin when pre- vs. post- treatment levels were compared using a one-tailed *t*-test (*p* < 0.05; Supplementary Table S1). To eliminate large-scale false discovery, a multiple hypothesis testing correction was applied using the Hochberg adjustment method available through an online analytics tool: https://multipletesting.com. After performing the Hochberg multiple hypothesis testing correction, seven miRNAs (hsa-miR-374a-5p, hsa-miR-640, hsa-miR-301b-3p, hsa-miR-1272, hsa-miR-3613-3p, hsa-miR-4531, hsa-miR-874-3p) were identified as being significantly reduced by GNA affinity resin treatment (Table 2).

**Figure 3:**
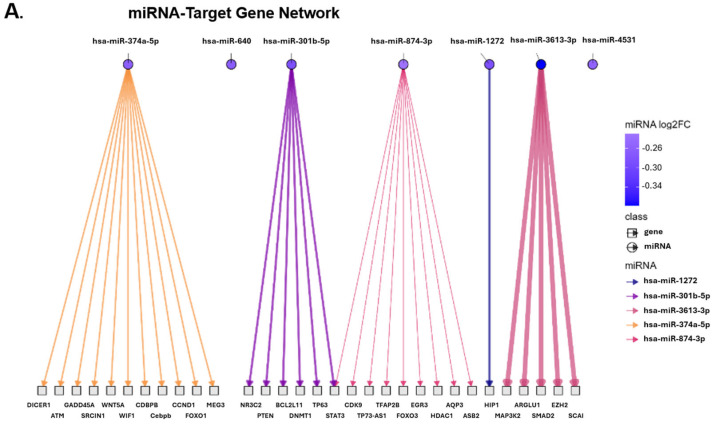

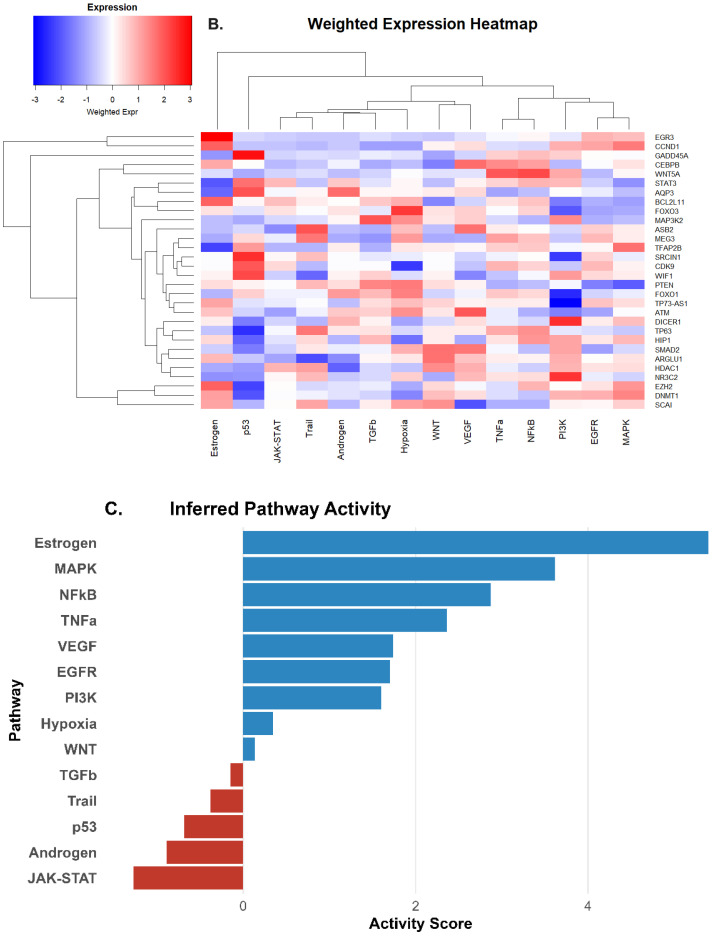
Computational miRNA pathway inference. (**A**) Sugiyama Framework plot depicting experimentally validated target genes of the 7 miRNAs significantly reduced by GNA resin treatment. Line thickness corresponds to the magnitude of the miRNA log2 (fold change). (**B**) Weighted Expression Heatmap depicting relevance of potential target gene changes on signaling pathways (blue = suppressed; white = unchanged; red = de-repressed). (**C**) Inferred Activity Score of canonical signaling pathway changes generated from the computational model.

To explore the potential effects of the reduction of these 7 miRNAs, we selected a methodology to expand our understanding of their mRNA targets. We used miRTarBase to retrieve evidence of experimentally validated miRNA-target interactions. As shown in Figure 3A, we identified a total of 32 gene transcripts targeted by one or more of these miRNAs. We then used the PROGENy pathway analysis tool to infer how potential changes in activity of these genes could relate to 14 well-established canonical signaling pathways. For this analysis, we assumed that the reduction of a miRNA suppressor could result in an equal proportional uptick in target gene expression. A PROGENy weighted-expression heatmap was created to depict how these modeled target-gene changes could affect the core signaling pathways (Figure 3B). Lastly, an activity score assigned to each pathway suggested that, under this model, the Estrogen pathway had the highest positive inferred score, while JAK-STAT had the most negative inferred score (Figure 3C).

## 3. Discussion

We found substantial variability in plasma EV populations during the post-acute phase of COVID-19, with some EV characteristics differing between individuals with Long COVID and those who fully recovered from their infection. Mannose-positive large EVs were significantly increased in Long COVID plasma compared with recovered controls, whereas total large EV and sEV quantities showed substantial variability and did not differ significantly between groups. We also observed that GNA affinity resin captured a measurable fraction of sEVs and that sEV capture showed a weak but significant association with mannose-positive large EV abundance in All LC samples. These findings support the presence of altered mannose-associated EV features in Long COVID plasma and provide a rationale for additional studies using GNA lectin to capture these EVs.

Even though there is limited literature directly assessing mannosylation on EVs in acute SARS-CoV-2 infection or Long COVID, there is evidence suggesting that the glycosylation pathway of the host during acute infection is altered. SARS-CoV-2 spike contains many glycosylation sites, several of which consistently retain high-mannose glycans [18]. Viral entry into ACE2-expressing host cells requires spike, and both viral and host glycoproteins utilize the endoplasmic reticulum (ER) and Golgi glycosylation machinery [19,20]. Genome-wide CRISPR screens and perturbation studies have identified N-glycosylation enzymes as essential host dependency factors for SARS-CoV-2 infection [21,22]. Clinical glycomics analyses of acute COVID-19 further demonstrate increased high-mannose content on immunoglobulins, particularly IgM, which correlates with disease severity [23]. Evidence from other diseases further supports the biological relevance of EV glycosylation in disease pathogenesis. High-mannose glycans have been identified on cancer-derived EVs and used for lectin-based EV capture, while glycan patterns on EVs released from Plasmodium falciparum-infected human erythrocytes facilitate uptake by human monocytes [13,14]. These findings demonstrate that disease-associated EV glycan patterns can influence vesicle recognition, isolation, and immune-cell interactions. Nevertheless, little is known about the cargo or functional consequences of EVs displaying altered mannosylation in Long COVID or other diseases. Our finding of increased mannosylation on plasma EVs therefore identifies a previously under characterized EV surface feature in Long COVID and provides a rationale for further characterization of GNA-binding EV populations, their associated cargo, and their potential biological activity.

In Long COVID, circulating dysregulated miRNA profiles can also contribute to pathogenic processes including inflammation and immune dysregulation [24]. Similarly, in acute COVID-19, specific miRNA enriched within circulating extracellular vesicles (EV-miRNA) have been shown to participate in proinflammatory and prothrombotic complications [25]. In the present study, GNA affinity resin treatment was significantly associated with reduction of seven circulating plasma miRNAs after multiple-testing correction. However, because miRNAs were measured in plasma before and after resin treatment rather than purified GNA-captured EVs, the present data does not establish that these miRNAs were exclusively EV-associated or located within mannose-positive EVs. Therefore, these findings are best interpreted as plasma miRNA changes after GNA resin treatment, rather than definitive evidence of selective EV-miRNA removal.

The seven miRNAs significantly reduced after GNA treatment allowed us to generate hypotheses regarding potential downstream signaling networks (Table 2, Figure 3). In this computational model, the strongest negative inferred activity score was observed for the JAK-STAT pathway, while Estrogen, VEGF, EGFR, and PI3K showed positive inferred activity scores. These pathway-level predictions are biologically relevant to Long COVID because persistent interferon signaling, immune dysregulation, endothelial dysfunction, and altered tissue-repair responses have been reported in subsets of patients [26,27,28,29,30,31]. The inferred reduction in JAK-STAT activity is notable in this context, as sustained interferon-associated signaling has been proposed to contribute to persistent symptoms, and JAK-STAT pathway inhibition is currently being investigated in Long COVID clinical trials involving neurocognitive and cardiopulmonary symptoms (NCT05858515, NCT06597396, NCT06928272). Furthermore, the inferred increase in Estrogen, VEGF, EGFR, and PI3K pathways may be relevant to vascular and endothelial dysfunction in Long COVID as the activation of these signaling networks are known to provide microvascular support, nitric oxide-mediated vasodilation, and endothelial repair [27,28,29]. On the other hand, activation of pathways such as NF-kB, MAPK, and TNF-α is generally linked to the amplification of inflammatory cytokines [30]. Therefore, inferred activation of such pathways might seem counterintuitive given that subsets of Long COVID patients have persistent inflammatory activity. However, multi-omics data demonstrates that Long COVID is clinically and biologically heterogeneous, identifying patient subsets with suppressed NF-kB signaling and impaired immune responsiveness [31]. In that context, an inferred uptick of NF-kB, MAPK, and TNF-α signaling could represent a candidate pathway pattern worth further investigation. However, these interpretations should be considered hypothesis-generating, as the pathway analysis was based on miRNA-target relationships and modeled pseudo-expression values rather than measured target-gene expression, protein activity, or functional cellular assays. Overall, these computational findings identify signaling axes that may be biologically relevant to Long COVID and should be validated in future studies.

The observation that sEV capture was associated with mannose-positive large EV measurements in All LC participants suggests that GNA-binding glycosylation may be detectable across more than one EV size subpopulation. This finding is translationally meaningful because it links a disease-associated EV glycosylation phenotype in Long COVID plasma with a defined lectin-based capture mechanism. Rather than relying only on theoretical disease associations, these data provide experimental evidence that glycosylated EV-associated plasma components can be captured by GNA affinity resin and may therefore represent a measurable and targetable circulating feature of Long COVID. Given prior reports implicating EVs as carriers of viral antigens, inflammatory signals, and other disease-relevant cargo in Long COVID, GNA-binding EV populations warrant further investigation as potential contributors to persistent immune activation, endothelial signaling abnormalities, viral antigen carriage, autoantibody-associated material, or other Long COVID-relevant phenotypes [10,32]. Future functional and clinical studies will be important to determine whether GNA-based extracorporeal capture can modify these biological processes in a manner relevant to Long COVID pathophysiology.

This study has several limitations. The small sample size in this exploratory pilot study limits the statistical power and the generalizability of subgroup comparisons. The study was cross-sectional, and the timing of samples studied was relatively early in the post-acute phase, slightly before the recently defined 90-day threshold for Long COVID. Potential confounding variables, including BMI, comorbidities, medication use, acute disease severity, time from infection, and symptom heterogeneity, may influence circulating EV and miRNA profiles. The NanoString nCounter platform utilizes an 827 predetermined human miRNA panel, excluding potential treatment relevant miRNA changes not part of the repertoire. The potential downstream effects of these miRNA reductions were computationally inferred using PROGENy. In this process, we mapped the miRNA-mRNA interactions from miRTarBase using only strong-evidence interactions, thus increasing the confidence of our inferred miRNA effects. Nevertheless, this approach might also be excluding less well-characterized miRNA-mRNA interactions. We also did not interrogate the mannosylated EVs for SARS-CoV-2 particles, autoantibodies or reactivated EBV which have been implicated in the pathogenesis of Long COVID. These experiments would be the next pre-clinical step in considering the potential utility of the Hemopurifier^®^ in Long COVID. Future studies should directly characterize GNA-captured material and test whether its removal alters Long COVID-relevant cellular responses. Ultimately, clinical studies using the Aethlon Hemopurifier^®^ with appropriate biomarker and symptom endpoints would be required to determine whether GNA-based extracorporeal capture has therapeutic relevance in this population.

## 4. Materials and Methods

### 4.1. Participant Selection and Evaluation

All samples were from individuals enrolled in the San Francisco-based Long-term Impact of Infection with Novel Coronavirus (LIINC) cohort (NCT04362150). Details of the cohort design have been previously published [33]. Briefly, adults with a history of test-confirmed SARS-CoV-2 infection are assessed at baseline and at approximately 3-month intervals thereafter, during which they undergo interviewer-administered assessments of COVID-attributed symptoms, quality of life, and medical comorbidities, as well as biospecimen collection. Following clinician review, participants are categorized as having Long COVID if they report symptoms that are new or worsened at least 90 days after an episode of COVID-19 that are not attributable to another cause; this approach is consistent with widely accepted definitions of Long COVID put forth by the World Health Organization (WHO) [34] and National Academies of Sciences, Engineering, and Medicine (NASEM) [35].

For this analysis, we studied frozen plasma samples from 15 participants in each of three clinically defined groups: fully recovered from prior SARS-CoV-2 infection (Recovered), Long COVID without neuropsychiatric symptoms (LC), and Long COVID with neuropsychiatric symptoms (NeuroLC). Neuropsychiatric symptoms included brain fog, headaches, dizziness, and difficulties with balance or vision. To compare all individuals experiencing any symptom persistence to those who recovered, we combined LC and NeuroLC participants into one group (All LC).

### 4.2. Patient Consent Statement

This research was approved by the UCSF Institutional Review Board (IRB), and all participants provided written informed consent.

### 4.3. Isolation of Large EVs

Plasma sample aliquots of 1 mL were precleared by centrifuging twice at 2500× *g* for 15 min at room temperature to pellet platelets and large cellular debris. The supernatant recovered from each spin was collected carefully to avoid disturbing the pellet. This precleared plasma was then centrifuged at 18,000× *g* for 45 min at 4 °C to pellet larger extracellular particles. The 18,000× *g* supernatant was completely removed and stored at −80 °C for downstream assays. The remaining pellet was resuspended in 100 µL of 0.22 µm PES-filtered PBS by rigorous pipetting after incubation at room temperature for 45 min and stored at −80 °C in 25 µL aliquots for downstream nanoparticle flow cytometry analysis.

### 4.4. Isolation of sEVs

Purification of sEVs was achieved by thawing a 150 µL 18,000× *g* precleared plasma aliquot from each participant and loading it into a qEV 35 nm Single-Use Gen2 size-exclusion chromatography (SEC) column (IZON, Medford, MA, USA, #ICS35-1381) containing a Sepharose bead bed. Equilibration of the column, flushing, and purified sEV fraction collection were performed using 0.22 µm PES-filtered PBS following standard indications on the Automatic Fraction Collector (IZON, Medford, MA, USA, #AFC-V2). A purified sEV solution of ~370 µL was collected from each participant’s plasma sample and then stored at −80 °C for downstream assays.

### 4.5. EV Quantification and Characterization

Extracellular vesicles were stained with MemGlow638nm (Cytoskeleton, Denver, CO, USA, #MG04-10), a phospholipid bilayer dye, incubated for one hour at room temperature, and quantified using the single-particle detection Flow NanoAnalyzer U30 instrument (NanoFCM, Inc., Xiamen, China). The instrument was calibrated using standard fluorescent 250 nm silica beads of known nanoparticle concentration (NanoFCM, Las Vegas, NV, USA, #QS2503). Size distribution profiles were established using the manufacturer’s calibration kits with mixtures of 155, 270, 535, and 850 nm silica beads (NanoFCM, Las Vegas, NV, USA, #S17M-MV) for characterization of the 18,000× *g*-enriched EVs, which we refer to as “large EVs”. These large EVs were characterized for surface mannose by staining and incubating at room temperature for one hour with the fluorescently labeled, soluble GNA (EY Labs, San Mateo, CA, USA, SKU#F-7401-2) in combination with MemGlow638nm and gated in the 100–500 nm diameter range. For flow nanoanalysis of SEC-isolated small EVs, a particle size distribution curve was established using a silica bead mixture containing 68, 91, 113, and 155 nm size nanoparticles (NanoFCM, Las Vegas, NV, USA, #S16M-Exo). We refer to our SEC-isolated EVs as “small EVs” or “sEVs,” which are measured in the 40–200 nm particle diameter window and stained for the presence of phospholipid bilayer with the MemGlow638nm dye (Cytoskeleton, Denver, CO, USA, #MG04-10).

### 4.6. sEV Capture Assay

Two paired 150 µL aliquots of isolated sEVs from the LIINC plasma samples were compared to assess sEV capture by the GNA affinity resin. One aliquot was incubated with 20 ± 0.2 mg of GNA affinity resin (AEMD, San Diego, CA, USA, Lot#AMI24002-023) for 1 h at room temperature on the RotoFlex at 40 rpm, together with its paired control aliquot lacking GNA resin. After treatment, the samples were briefly spun at 1000× *g* for 5 min to clarify the supernatant, then the sEV quantities were measured using the Flow NanoAnalyzer instrument. Differences in sEV content between the paired samples were attributed to sEV binding and capture on the GNA affinity resin.

### 4.7. miRNA Capture Assay

In this stage, 330 µL aliquots of 18,000× *g*-clarified plasma from All LC participants were treated with 44 ± 0.4 mg of GNA affinity resin (AEMD, Lot#AMI24002-023) by mixing for one hour at room temperature on the RotoFlex at 40 rpm together with its untreated paired control. After treatment, the samples were briefly spun for 5 min at 1000× *g* to pellet any resin fines. RNA was isolated from 200 µL of supernatant using the miRNeasy Serum/Plasma Advanced kit (Qiagen, Germantown, MD, USA, #217204) with the addition of 5 µL of a 200 pM mixture of three exogenous miRNAs (ath-miR159a, cel-miR-248, osa-miR414) to facilitate downstream analysis. Purified miRNA was eluted into 14 µL of RNase-free water and sent for external analysis to Bruker Spatial Biology for evaluation with the off-the-shelf NanoString nCounter miRNA expression panel (Human v3: CSO-MIR3-12), which measures the content of 827 different human miRNAs as well as the exogenous miRNA controls. Data were collected with the nCounter MAX Analysis system (Bruker Spatial Biology, Bothell, WA, USA).

### 4.8. NanoString Data Analysis

The NanoString nCounter analysis produced raw count data for each miRNA detected by the expression panel. The data were assessed using the nSolver 4.0 software to set a background threshold equal to the maximum output of the negative controls and normalized to the geometric mean of the three positive ligation controls and two of the exogenous spike-in miRNAs (cel-miR-248 and osa-miR414). We noted that the exogenous ath-miR159a spike-in miRNA failed to reach the detection threshold in all samples and was therefore excluded as normalization control. Additional data quality assessments identified specific samples in which either the positive ligation controls or the functional exogenous spike-in miRNAs had deficient detection signals, and these were excluded from further comparative analysis. In total, 20 paired plasma samples from All LC participants (*n* = 9 LC + *n* = 11 NeuroLC) satisfied all quality control criteria and were used to evaluate changes in miRNA content following GNA affinity resin treatment.

### 4.9. Pathway Activity Inference

To estimate pathway activity changes downstream of miRNA reduction, we implemented a custom workflow integrating validated miRNA-mRNA interactions with footprint-based pathway models. miRNA-target interactions were obtained from miRTarBase (Release 10), using entries annotated as strong-evidence interactions [36]. For each target gene, “pseudo-expression” values were simulated under the assumption that downregulated miRNAs lead to relative de-repression of their target transcripts. When multiple miRNAs targeted the same gene, effects were aggregated using a weighted mean across interactions. These pseudo-expression profiles were analyzed using the PROGENy R package (v1.26.0, Bioconductor) to infer changes across fourteen canonical signaling pathways [37]. Pathway activity scores were obtained using all footprint genes available to maximize coverage. Directed miRNA-mRNA interactions were visualized using a layered Sugiyama layout implemented with the ggraph package (v2.2.1). A weighted expression heatmap was generated using the pheatmap package (v1.0.13) and a bar plot of inferred pathway activity was produced with ggplot2 package (v3.5.2). All analyses were conducted in R (v4.4.1).

### 4.10. Statistical Analysis

Descriptive statistics were used to summarize clinical characteristics. Parametric analyses were applied to evaluate plasma EV characteristics and contents. A one-tailed t-test assuming unequal variance was used to compare EV quantities and surface glycan characteristics between the symptom groups and the control group, with *p* < 0.05 considered significant. Pearson’s correlation coefficient was used to examine pairwise relationships and to compare both EV quantities and mannose-positive GNA binding characteristics among distinct EV subpopulations. These analyses and graphs were generated using Excel (version 2507) and GraphPad Prism 10.6.0. To identify miRNAs significantly reduced by GNA resin treatment, normalized NanoString data were analyzed with a one-tailed *t*-test in R (v4.4.1) with *p* < 0.05 considered significant. A more conservative Hochberg correction was subsequently applied to adjust for multiple hypothesis testing and increase confidence in identifying the plasma miRNAs significantly reduced by treatment using the online calculator (https://www.multipletesting.com/analysis/, accessed on 1 August 2025) [38].

## Figures and Tables

**Table 1:** Demographic and clinical characteristics of participant groups. Participants fully recovered from COVID (Recovered), participants with Long COVID (LC), and symptomatic Long COVID with neurological complications (NeuroLC). All LC includes all Long COVID symptoms (LC + NeuroLC).

Characteristic	Participant Symptom Group			
	Recovered (*n* = 15)	LC (*n* = 15)	NeuroLC (*n* = 15)	All LC (*n* = 30)
Age, median [IQR]	37 [30–48]	48 [32–51]	38 [34–46]	40 [34–49]
Sex at birth				
Female, *n* (%)	8 (53.3)	9 (60)	9 (60)	18 (60)
Male, *n* (%)	7 (46.7)	6 (40)	6 (40)	12 (40)
Race/ethnicity				
Asian, *n* (%)	4 (26.7)	1 (6.7)	1 (6.7)	2 (6.7)
Hispanic/Latino, *n* (%)	5 (33.3)	4 (26.7)	7 (46.7)	11 (36.7)
Black/African American, *n* (%)	0 (0)	3 (20)	0 (0)	3 (10)
White, *n* (%)	5 (33.3)	7 (46.7)	7 (46.7)	14 (46.7)
Declined to answer, *n* (%)	1 (6.7)	0 (0)	0 (0)	0 (0)
Hospitalized during acute COVID-19	5 (33.3)	4 (26.7)	7 (46.7)	11 (36.7)
BMI				
<25	8 (53.3)	4 (26.7)	5 (33.3)	9 (30)
25–30	5 (33.3)	7 (46.7)	6 (40)	13 (43.3)
>30	2 (13.3)	4 (26.7)	4 (26.7)	8 (26.7)
Medical history				
Autoimmune disease, *n* (%)	1 (6.7)	1 (6.7)	2 (13.3)	3 (10)
Cancer treated within past 2 years, *n* (%)	0 (0)	1 (6.7)	0 (0)	1 (3.3)
Diabetes, *n* (%)	3 (20)	1 (6.7)	1 (6.7)	2 (6.7)
Heart attack or heart failure, *n* (%)	0 (0)	0 (0)	0 (0)	0 (0)
Hypertension or high blood pressure, *n* (%)	0 (0)	3 (20)	3 (20)	6 (20)
Lung disease, *n* (%)	2 (13.3)	5 (33.3)	1 (6.7)	6 (20)
Kidney disease, *n* (%)	0 (0)	1 (6.7)	0 (0)	1 (3.3)
Long COVID symptoms, median (IQR)	0 [0]	3 [1–3]	5 [3–8]	3 [3–5]
Days post symptom onset, median (IQR)	72 [68–88]	77 [62–86]	78 [73–81]	78 [66–83]

Abbreviations: BMI, body mass index (weight in kg divided by the height in meters squared); IQR, interquartile range [25th–75th percentile]; *n* (%), number and percentage of participants in the corresponding group.

**Table 2:** miRNAs significantly reduced by GNA affinity resin treatment in All LC plasma samples.

miRNA	Accession #	Log2 Fold Change	Percent Reduction
hsa-miR-374a-5p	MIMAT0000727	−0.26	−19.9%
hsa-miR-640	MIMAT0003310	−0.27	−20.3%
hsa-miR-301b-3p	MIMAT0004958	−0.29	−21.9%
hsa-miR-1272	MIMAT0005925	−0.29	−22.0%
hsa-miR-3613-3p	MIMAT0017991	−0.38	−30.0%
hsa-miR-4531	MIMAT0019070	−0.26	−19.4%
hsa-miR-874-3p	MIMAT0004911	−0.23	−17.3%

## Data Availability

The raw data supporting the conclusions of this article will be made available by the authors on request.

## Supplementary Materials

The following supporting information can be downloaded at: https://www.mdpi.com/article/10.3390/ijms27135723/s1.

## Abbreviations

The following abbreviations are used in this manuscript (arranged alphabetically):

µL	microliter
µm	micrometer
ACE2	Angiotensin-Converting Enzyme 2
All LC	All Long COVID (neuropsychiatric and non-neuropsychiatric symptom groups combined)
BMI	Body Mass Index
°C	degree Celsius
COVID	Coronavirus Disease
CRISPR	Clustered Regularly Interspaced Short Palindromic Repeats
EGFR	Epidermal Growth Factor Receptor
EVs	Extracellular Vesicles
GNA	*Galanthus nivalis* agglutinin
IgM	Immunoglobulin M
IQR	Interquartile Range
JAK-STAT	Janus Kinase—signal transducer and activator of transcription
LC	Long COVID (non-neuropsychiatric symptom group)
LIINC	Long-term Impact of Infection with Novel Coronavirus
MAPK	Mitogen-Activated Protein Kinase
miRNA	Micro RNA
mL	milliliter
mRNA	messenger RNA
*n*	number of samples or participants
NeuroLC	Neuropsychiatric Long COVID (neuropsychiatric symptom group)
NF-kB	Nuclear Factor kappa-light-chain-enhancer of activated B cells
nm	nanometer
PES	Polyethersulfone
PI3K	Phosphatidylinositol 3-Kinase
pM	picomolar
RNA	Ribonucleic Acid
SARS-CoV-2	Severe Acute Respiratory Syndrome Coronavirus 2
SD	Standard Deviation
SEC	Size Exclusion Chromatography
sEV	small EVs
TNF-α	Tumor Necrosis Factor-alpha
VEGF	Vascular Endothelial Growth Factor
