# Supplementary material for: Increased Mannosylation of Extracellular Vesicles in Long COVID Plasma as a Binding Target for Galanthus nivalis Agglutinin (GNA) Affinity Resin
Source: Int J Mol Sci. 2026 Jun 25;27(13):5723. doi: 10.3390/ijms27135723 (PMC13361104; doi:10.3390/ijms27135723)
Supplement: Supplementary file 1 [file ijms-27-05723-s001.zip › ijms-4330229-supplementary.pdf]

Supplementary Table S1. List of miRNAs reduced by GNA resin treatment in All LC plasma samples.

miRNA	Accession #	Log_2 (fold change)	p value
hsa-miR-374a-5p	MIMAT0000727	-0.26	0.002
hsa-miR-640	MIMAT0003310	-0.27	0.002
hsa-miR-301b-3p	MIMAT0004958	-0.29	0.003
hsa-miR-1272	MIMAT0005925	-0.29	0.005
hsa-miR-3613-3p	MIMAT0017991	-0.38	0.005
hsa-miR-4531	MIMAT0019070	-0.26	0.005
hsa-miR-874-3p	MIMAT0004911	-0.23	0.007
hsa-miR-320e	MIMAT0015072	-0.27	0.007
hsa-miR-590-5p	MIMAT0003258	-0.23	0.007
hsa-miR-455-3p	MIMAT0004784	-0.23	0.007
hsa-miR-371a-5p	MIMAT0004687	-0.41	0.007
hsa-miR-1183	MIMAT0005828	-0.23	0.007
hsa-miR-133a-3p	MIMAT0000427	-0.31	0.008
hsa-miR-210-3p	MIMAT0000267	-0.38	0.008
hsa-miR-652-5p	MIMAT0022709	-0.23	0.008
hsa-miR-2110	MIMAT0010133	-0.30	0.008
hsa-miR-4707-5p	MIMAT0019807	-0.24	0.008
hsa-miR-502-3p	MIMAT0004775	-0.22	0.008
hsa-miR-574-3p	MIMAT0003239	-0.27	0.009
hsa-miR-1286	MIMAT0005877	-0.23	0.009
hsa-miR-587	MIMAT0003253	-0.21	0.009
hsa-miR-513a-3p	MIMAT0004777	-0.27	0.010
hsa-miR-182-3p	MIMAT0000260	-0.22	0.010
hsa-miR-1257	MIMAT0005908	-0.22	0.010
hsa-miR-20a-5p+hsa-miR-20b-5p	MIMAT0000075	-0.23	0.010
hsa-miR-1304-5p	MIMAT0005892	-0.35	0.010
hsa-miR-3180-3p	MIMAT0015058	-0.25	0.010
hsa-miR-219b-3p	MIMAT0019748	-0.24	0.010
hsa-miR-328-5p	MIMAT0026486	-0.28	0.010
hsa-miR-548ak	MIMAT0019013	-0.23	0.011
hsa-miR-556-3p	MIMAT0004793	-0.23	0.011
hsa-miR-520f-3p	MIMAT0002830	-0.21	0.011
hsa-miR-128-1-5p	MIMAT0026477	-0.22	0.011
hsa-miR-135b-5p	MIMAT0000758	-0.22	0.011
hsa-miR-549a	MIMAT0003333	-0.34	0.012
hsa-miR-34a-5p	MIMAT0000255	-0.23	0.012

hsa-miR-1537-3p	MIMAT0007399	-0.26	0.012
hsa-miR-660-5p	MIMAT0003338	-0.23	0.012
hsa-miR-19b-3p	MIMAT0000074	-0.29	0.012
hsa-miR-412-3p	MIMAT0002170	-0.25	0.013
hsa-miR-1307-5p	MIMAT0022727	-0.21	0.013
hsa-miR-190a-3p	MIMAT0026482	-0.21	0.013
hsa-miR-503-3p	MIMAT0022925	-0.21	0.013
hsa-miR-6511a-3p	MIMAT0025479	-0.21	0.013
hsa-miR-499b-3p	MIMAT0019898	-0.21	0.013
hsa-miR-23c	MIMAT0018000	-0.41	0.014
hsa-miR-4755-5p	MIMAT0019895	-0.22	0.014
hsa-miR-23b-3p	MIMAT0000418	-0.27	0.014
hsa-miR-1973	MIMAT0009448	-0.25	0.014
hsa-miR-1307-3p	MIMAT0005951	-0.21	0.014
hsa-miR-509-3p	MIMAT0002881	-0.21	0.014
hsa-miR-19a-3p	MIMAT0000073	-0.22	0.014
hsa-miR-3185	MIMAT0015065	-0.21	0.015
hsa-miR-570-3p	MIMAT0003235	-0.25	0.015
hsa-miR-936	MIMAT0004979	-0.21	0.015
hsa-miR-18b-5p	MIMAT0001412	-0.21	0.016
hsa-miR-3161	MIMAT0015035	-0.21	0.016
hsa-miR-487b-3p	MIMAT0003180	-0.21	0.016
hsa-miR-499a-5p	MIMAT0002870	-0.21	0.016
hsa-miR-524-3p	MIMAT0002850	-0.21	0.016
hsa-miR-3613-5p	MIMAT0017990	-0.21	0.016
hsa-miR-631	MIMAT0003300	-0.21	0.016
hsa-miR-4787-5p	MIMAT0019956	-0.21	0.016
hsa-miR-455-5p	MIMAT0003150	-0.27	0.016
hsa-miR-1203	MIMAT0005866	-0.24	0.016
hsa-miR-876-5p	MIMAT0004924	-0.22	0.016
hsa-miR-217	MIMAT0000274	-0.25	0.017
hsa-miR-548m	MIMAT0005917	-0.34	0.018
hsa-miR-423-3p	MIMAT0001340	-0.21	0.018
hsa-let-7c-5p	MIMAT0000064	-0.21	0.018
hsa-miR-125b-5p	MIMAT0000423	-0.21	0.018
hsa-miR-365b-5p	MIMAT0022833	-0.21	0.018
hsa-miR-221-3p	MIMAT0000278	-0.21	0.018
hsa-miR-302a-5p	MIMAT0000683	-0.21	0.018
hsa-miR-1193	MIMAT0015049	-0.20	0.019

hsa-miR-1205	MIMAT0005869	-0.20	0.019
hsa-miR-1258	MIMAT0005909	-0.20	0.019
hsa-miR-1264	MIMAT0005791	-0.20	0.019
hsa-miR-1322	MIMAT0005953	-0.20	0.019
hsa-miR-143-3p	MIMAT0000435	-0.20	0.019
hsa-miR-224-5p	MIMAT0000281	-0.20	0.019
hsa-miR-302f	MIMAT0005932	-0.20	0.019
hsa-miR-3180	MIMAT0018178	-0.20	0.019
hsa-miR-516a-3p+hsa-miR-516b-3p	MIMAT0006778	-0.20	0.019
hsa-miR-518f-3p	MIMAT0002842	-0.20	0.019
hsa-miR-541-3p	MIMAT0004920	-0.20	0.019
hsa-miR-542-5p	MIMAT0003340	-0.20	0.019
hsa-miR-564	MIMAT0003228	-0.20	0.019
hsa-miR-644a	MIMAT0003314	-0.20	0.019
hsa-miR-940	MIMAT0004983	-0.20	0.019
hsa-miR-1224-3p	MIMAT0005459	-0.20	0.019
hsa-miR-1261	MIMAT0005913	-0.20	0.019
hsa-miR-141-3p	MIMAT0000432	-0.20	0.019
hsa-miR-147b	MIMAT0004928	-0.20	0.019
hsa-miR-506-3p	MIMAT0002878	-0.20	0.019
hsa-miR-612	MIMAT0003280	-0.28	0.019
hsa-miR-2113	MIMAT0009206	-0.21	0.019
hsa-miR-181a-5p	MIMAT0000256	-0.21	0.020
hsa-miR-449a	MIMAT0001541	-0.20	0.020
hsa-miR-411-5p	MIMAT0003329	-0.23	0.020
hsa-miR-1245a	MIMAT0005897	-0.25	0.021
hsa-miR-502-5p	MIMAT0002873	-0.32	0.022
hsa-miR-517c-3p+hsa-miR-519a-3p	MIMAT0002866	-0.29	0.023
hsa-miR-556-5p	MIMAT0003220	-0.22	0.023
hsa-miR-376a-2-5p	MIMAT0022928	-0.19	0.024
hsa-miR-128-3p	MIMAT0000424	-0.19	0.025
hsa-miR-134-3p	MIMAT0026481	-0.24	0.025
hsa-miR-208a-3p	MIMAT0000241	-0.25	0.026
hsa-miR-92b-3p	MIMAT0003218	-0.19	0.026
hsa-miR-6720-3p	MIMAT0025851	-0.27	0.026
hsa-miR-345-3p	MIMAT0022698	-0.22	0.027
hsa-miR-106a-5p+hsa-miR-17-5p	MIMAT0000103	-0.24	0.028
hsa-miR-548al	MIMAT0019024	-0.26	0.030
hsa-miR-603	MIMAT0003271	-0.65	0.030

hsa-miR-302a-3p	MIMAT0000684	-0.19	0.031
hsa-miR-656-3p	MIMAT0003332	-0.18	0.031
hsa-miR-342-3p	MIMAT0000753	-0.19	0.033
hsa-miR-24-3p	MIMAT0000080	-0.24	0.035
hsa-miR-4461	MIMAT0018983	-0.27	0.037
hsa-miR-582-5p	MIMAT0003247	-0.25	0.039
hsa-miR-29c-3p	MIMAT0000681	-0.18	0.043
hsa-miR-29a-3p	MIMAT0000086	-0.22	0.043
hsa-miR-299-5p	MIMAT0002890	-0.25	0.045
hsa-miR-548aa+hsa-miR-548t-3p	MIMAT0018447	-0.39	0.046
hsa-miR-496	MIMAT0002818	-0.28	0.047
hsa-miR-1278	MIMAT0005936	-0.20	0.048
hsa-miR-548v	MIMAT0015020	-0.29	0.048
